# Myosin and $\upalpha$-actinin regulation of stress fiber contractility under tensile stress

**DOI:** 10.1038/s41598-023-35675-7

**Published:** 2023-05-29

**Authors:** Haoran Ni, Qin Ni, Garegin A. Papoian, Andreea Trache, Yi Jiang

**Affiliations:** 1 Institute for Physical Science and Technology, University of Maryland, College Park, MD, USA; 2 Institute for NanoBioTechnology, Johns Hopkins University, Baltimore, MD, USA; 3 Department of Chemistry and Biochemistry, University of Maryland, College Park, MD, USA; 4 Department of Medical Physiology, Texas A &M University Health Science Center, Bryan, TX, USA; 5 Department of Biomedical Engineering, Texas A &M University, College Station, TX, USA; 6 Department of Mathematics and Statistics, Georgia State University, Atlanta, GA, USA

**Keywords:** Stress fibres, Computational biophysics

## Abstract

Stress fibers are actomyosin bundles that regulate cellular mechanosensation and force transduction. Interacting with the extracellular matrix through focal adhesion complexes, stress fibers are highly dynamic structures regulated by myosin motors and crosslinking proteins. Under external mechanical stimuli such as tensile forces, the stress fiber remodels its architecture to adapt to external cues, displaying properties of viscoelastic materials. How the structural remodeling of stress fibers is related to the generation of contractile force is not well understood. In this work, we simulate mechanochemical dynamics and force generation of stress fibers using the molecular simulation platform MEDYAN. We model stress fiber as two connecting bipolar bundles attached at the ends to focal adhesion complexes. The simulated stress fibers generate contractile force that is regulated by myosin motors and $\alpha$-actinin crosslinkers. We find that stress fibers enhance contractility by reducing the distance between actin filaments to increase crosslinker binding, and this structural remodeling ability depends on the crosslinker turnover rate. Under tensile pulling force, the stress fiber shows an instantaneous increase of the contractile forces followed by a slow relaxation into a new steady state. While the new steady state contractility after pulling depends only on the overlap between actin bundles, the short-term contractility enhancement is sensitive to the tensile pulling distance. We further show that this mechanical response is also sensitive to the crosslinker turnover rate. Our results provide new insights into the stress fiber mechanics that have significant implications for understanding cellular adaptation to mechanical signaling.

## Introduction

In adherent cells, actin filaments bundle together to form stress fibers that regulate cell adhesion, migration, and mechanotransduction^1–4^. Originating from focal adhesion (FA) sites, dorsal stress fibers grow and bundle with each other through crosslinking proteins and molecular motors, forming ventral stress fibers connecting distant FA sites^2^. Recent experimental and theoretical efforts show that stress fiber can also generate from other actin structures such as the actin cortex^5,6^ and random actin networks^7,8^. Stress fibers are highly dynamic actomyosin structures regulated by myosin motors and crosslinkers. A ventral stress fiber usually displays mixed actin filament polarity to facilitate the sliding of myosin motors^2,9,10^, leading to contraction at the FA sites. At the same time, the crosslinking protein $\upalpha$-actinin works with myosin motors to modulate stress fiber bundling and contractility^2,11–13^. The dynamic turnover of $\upalpha$-actinin and its binding kinetics are sensitive to mechanical forces and other physiological conditions^14–19^. However, how the $\upalpha$-actinin turnover rate regulates the contractility of actomyosin stress fibers is not well understood.

Under biochemical and mechanical stimuli, cells adapt by reorganizing the ventral stress fibers, behaving like an active viscoelastic material^20–23^. External mechanical cues induce actin network remodeling, which alters the cell shape and its mechanical properties, through either signaling cascades or direct reshaping of network structures. For example, expanding cell surface via mechanical stretching or pulling activates calcium influx through mechanosensitive ion channel Piezo1^24–26^, leading to RhoA dependent myosin activation that enhances cellular contractility^27–31^. In stress fibers, the mechanosensory response of actomyosin networks through signaling cascades has been well established^1,3^. Actin networks can also sense the deformation and extracellular forces, and adapt directly by reorganizing actin, myosin motors, and crosslinkers^7,8,32,33^. How external mechanical stimuli induce enhanced contractility via stress fibers by structural remodeling is still unclear.

Inspired by actin remodeling experiments using traction force microscopy and atomic force microscopy^30,34–37^, in this work, we developed a molecular model to investigate the structural origin of mechanical forces in a stress fiber using the state-of-the-art mechano-chemical simulation platform MEDYAN^4,8,38^. We simulated a minimal stress fiber consisting of actin bundles anchored to two fixed focal adhesion sites, reproducing contractile force patterns resembling those observed experimentally in live cells^39^ and reconstituted actin bundles in vitro^40,41^. In addition, we also applied tensile forces to the stress fiber to study actin reorganization and contractility, reproducing experimental observations in cells that were stretched or pulled^30,34–37^. Our simple stress fiber model enabled a systematic exploration of the molecular determinants of contractile force generation. This study focused on myosin motors and crosslinkers, in particular, the concentrations of myosin and $\upalpha$-actinin proteins and the $\upalpha$-actinin turnover rate, with and without external tensile stress.

## Results

### A minimal molecular model of stress fiber contractility

**Figure 1:**
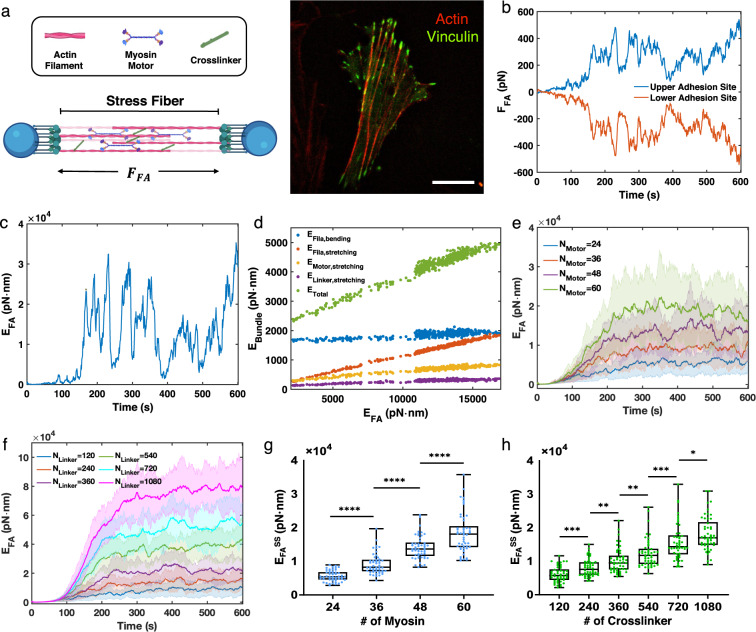
A minimal model of a stress fiber with molecular regulation. (**a**, left) The stress fiber is modeled as two actin bundles that are anti-parallel to each other. Each bundle is attached to the focal adhesion complex using elastic springs. Contractile forces are measured as the forces exerted on the focal adhesion sites. (**a**, right) A representative vascular smooth muscle cell expressing actin-mRFP (red) and vinculin-GFP (green) was plated on fibronectin and imaged by confocal microscopy. Scale bar is 20 µm. (**b,c**) The focal adhesion forces ($F_\text {FA}$, **b**) and the focal adhesion energy ($E_\text {FA}$, **c**) as a function of time from a representative simulation trajectory. (**d**) Relationship between $E_\text {FA}$ and mechanical energies of filament bending, filament stretching, myosin motor stretching, crosslinker stretching, and the total mechanical energy of the bundle, respectively. (**e,f**) The mean $E_\text {FA}$ as a function of time with varying numbers of myosin motors (**e**) and crosslinkers (**f**). Shading represents the 25th and 75th percentiles. (**g,h**) The steady-state focal adhesion energy ($E_\text {FA}^\text {SS}$, defined as the mean $E_\text {FA}$ after 300s) with varying numbers of myosin motors (**g**) and crosslinkers (**h**). In box plots, lower and upper boundaries represent the 25th and 75th percentiles, line inside box represents median, and lower and upper error lines represent the minimum and maximum values. Filled circles indicate all data points. The t-tests were performed, with ****p<0.0001, ***p<0.001,**p<0.01, and *p<0.05. (b-h) $N_\text {Motor} = 48$, $N_\text {Crosslinker} = 240$, and $\chi = 1$ unless otherwise noted.

We constructed a minimal model for stress fiber using MEDYAN. The key mechanochemical features include stretching and bending of actin filaments, binding and walking of myosin motors, binding and unbinding of crosslinkers, and the elastic attachments of the filaments to the focal adhesion complexes (Fig. 1a-left). Two focal adhesion sites were simulated as two beads with fixed positions. The actin filaments are attached to the focal adhesion sites via harmonic springs. This setup mimics the stress fibers formed between focal adhesion complexes in cells sitting on a substrate (Fig. 1a-right). We initialized unipolar, actin filament bundles, which resemble dorsal stress fibers, at the two focal adhesion sites. The actin filaments can polymerize towards each other. When they reach the steady state, the two bundles would overlap for approximately 600  nm, forming a minimal stress fiber with graded polarity (Supplementary Video 1). The plus ends of actin filaments were attached to the focal adhesion sites and the minus ends were pointing towards the other actin bundle. In this configuration, myosin motors can walk towards the plus ends and generate contraction. We simulated crosslinking proteins as harmonic springs that can connect between nearby actin filament pairs based on the properties of $\upalpha$-actinin. The model details can be found in the “Methods” section.

To quantitatively assess the mechanical properties of the simulated stress fiber, we measured the contractile force, or equivalently the collective stretching force, $F_\text {FA}$, between the focal adhesion site and the actin filaments attached to them (Fig. 1b). Our steady state contractile force, $F_\text {FA}$, has an order of magnitude of $10^{2}$ pN to $10^{3}$ pN, which is comparable to the contractile force measured in reconstituted stress fibers ($\sim {500}$ pN)^40^ and single stress fibers isolated from live cells ($\sim {10}$nN)^39^.

In addition, we measured the focal adhesion energy as the elastic energy between the focal adhesion sites and actin filaments attached to them ($E_\text {FA}$). This model allowed us to separately measure the individual contributions to the total mechanical energy of actin bundle $E_\text {bundle}$ ((Fig. 1c), from actin filaments, myosin motors, and crosslinkers, respectively (Fig. 1d). We found that $E_\text {FA}$ scales linearly with the total mechanical energy of stress fibers. The main contributor to the $E_\text {FA}$ is filament stretching, while motor and crosslinker stretching play relatively minor roles. Even though filament bending is the major contributor to $E_\text {bundle}$, it remains nearly constant regardless of the contractility. Therefore, we concluded that the $F_\text {FA}$ or $E_\text {FA}$ captures overall bundle contractile characteristics, and we henceforth used $E_\text {FA}$ to quantify the contractility generated from the simulated stress fibers.

Additionally, we varied the number of myosin motors and crosslinkers and examined the changes in contractility. The mean steady-state energy at FA sites, $E_\text {FA}^\text {SS}$, increases as the number of myosin and $\upalpha$-actinin proteins increase, which is consistent with previous findings from various actomyosin systems^13,42–51^ (Fig. 1e-h).

### Tight spacing between actin filaments promotes crosslinker binding and enhances contractility

**Figure 2:**
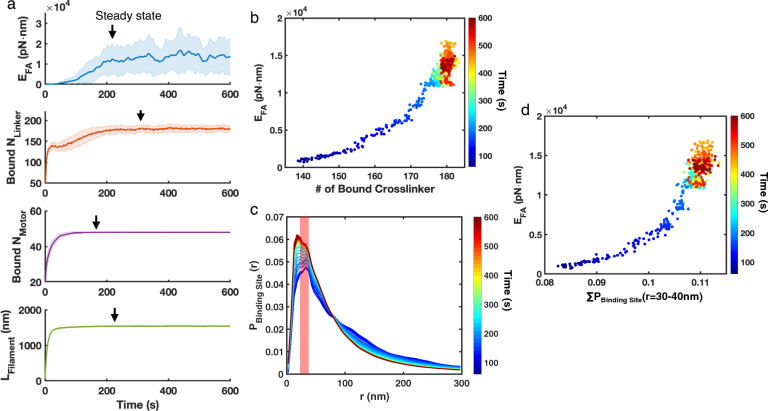
Stress fibers enhance contractility by promoting crosslinker binding during development. (**a**) The mean total focal adhesion energy, the number of bound crosslinkers, the number of bound motors, and the average filament length as a function of time. $E_\text {FA}$, the number of bound crosslinkers, the number of bound myosin motors, and the average filament length reach steady state at 210 s, 309 s, 171 s, and 212 s, respectively. Shading represents the 25th and 75th percentiles. (**b**) $E_\text {FA}$ correlates superlinearly with the number of bound crosslinkers during development. (**c**) The radial probability density distribution of the distance between two crosslinker binding sites ($P_\text {BindingSites}(r)$) during stress fiber development. The shaded pink region marks *r* corresponding to crosslinker binding ($r={30}$–40 nm, based on the length of $\upalpha$-actinin). Each line represents an average of 50 duplicated trajectories every 15 s. (**d**) $E_\text {FA}$ *versus* $P_\text {BindingSites}$ corresponding to crosslinker binding ($r={30}$–40 nm) during the stress fiber development. (**a–d**) $N_\text {Motor} = 48$ and $N_\text {Crosslinker} = 240$. n $= 50$ duplicated trajectories simulations.

During the stress fiber development, we found that contractility continuously increases till it reaches a steady state with a maximum value $E_\text {FA}$ at $\sim {210}$ s (Fig. 2a, see Method for details). Interestingly, the maximum crosslinker binding occurred at a later time ($\sim {309}$ s ) than the number of bound myosin motors ($\sim {171}$ s). These results suggest that the stress fiber is actively adjusting its chemical composition, mainly crosslinkers, to maximize the contractility. Indeed, $E_\text {FA}$ is positively associated with the number of bound crosslinkers during the stress fiber development, and the correlation is superlinear (Fig. 2b). After $\sim {171}$ s, the number of motors remains constant, while the number of bound crosslinkers keeps increasing, leading to a $\sim 2$-fold increase in $E_\text {FA}$.

One possible explanation is that stress fiber modifies its structure to optimize the spacing for crosslinker binding. Thus, we measured the radial probability density distribution of the distance between two crosslinker binding sites, $P_\text {BindingSites}(r)$ (Fig. 2c). We found that $P_\text {BindingSites}(r)$ peaks at around $r \sim 30$nm, which is close to the simulated length for crosslinkers (30–40 nm)^52^. In addition, the peak of $P_\text {BindingSites}(r)$ at $r \sim {30}$–40 nm continues to increase even after 310s, when the other measures have reached a steady state. As $P_\text {BindingSites}({30}-{40}$ nm) estimates the total available crosslinker binding sites, this observation also suggests that more potential binding sites could be created to further enhance crosslinking. We also showed that $E_\text {FA}$ is positively associated with the total $P_\text {BindingSites}(r)$ at the binding site length (30-40 nm), and the correlation is similar to that of the number of bound crosslinkers in a superlinear fashion (compare Fig. 2b, d). These observations suggest that before reaching maximum contractility, stress fibers “mature” by modulating the actin network structures to optimize crosslinker binding.

### A lower crosslinker turnover rate enhances stress fiber contractility and structural stability

**Figure 3:**
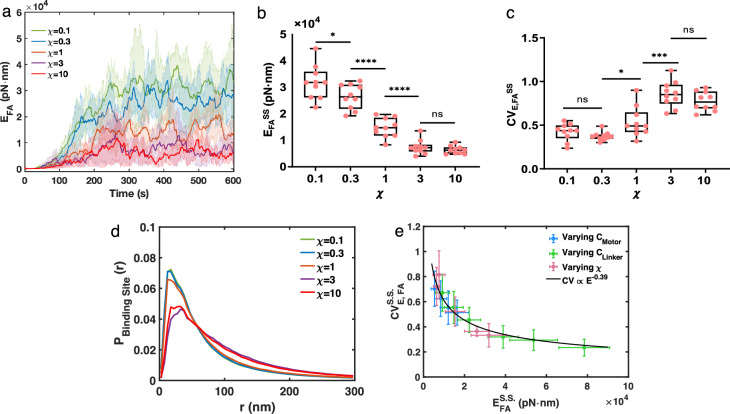
Stress fiber contractility and its fluctuations are regulated by crosslinker turnover. (**a–c**) Simulations with varying linker turnover rates. (**a**) The mean focal adhesion energy versus time. Shadings represent the 25th and 75th percentiles. $n = 10$ duplicated trajectories. (**b**) The steady-state focal adhesion energy decreases with a faster linker turnover rate. (**c**) The fluctuation of the focal adhesion energy (defined as CV) increases with a faster linker turnover rate. (**b,c**) In box plots, lower and upper boundaries represent 25th and 75th percentiles, the line inside the box represents the median, and the lower and upper error lines represent the minimum and maximum values. Filled circles indicate all data points. T-tests were performed, ****p<0.0001, ***p<0.001, *p<0.05, and ns p>0.05. (**d**) The steady state (300–600   s) $P_\text {BindingSites}(r)$ at different $\chi$. (**e**) $CV_\text {E, FA}^\text {SS}$ decreases with focal adhesion energies. The solid line is a power law fit (RMSE $=$ 0.05). Error bars represent the standard deviation over 10 (for varying $\chi$) or 50 (for varying motors or crosslinkers) duplicated trajectories. (**a–d**) $N_\text {Motor} = 48$ and $N_\text {Crosslinker} = 240$.

The binding kinetics of crosslinking proteins depend on the crosslinker type^14^, the mechanical force^15–17^, the physiological environment such as calcium concentration^18^, and their spatial localization^19^. Next, we explored how crosslinker binding kinetics affects stress fiber contractility by varying crosslinker turnover rates. To modulate the crosslinker turnover without changing the total number of bound crosslinkers, we scaled both the binding rate and the unbinding rate by a factor $\chi$. At the baseline $\chi =1$, crosslinkers have a lifetime of $\sim {3}$ s. Increasing $\chi$ reduces crosslinker turnover time. We used up to tenfold changes in the turnover rate to mimic those reported in different cell types and different actomyosin structures^14,19^. The result showed that changing $\chi$ has minimal impact on the number of crosslinkers bound to actin filaments (Supplementary Fig. S1). Interestingly, increasing the crosslinker turnover rate significantly reduces $E_\text {FA}^\text {SS}$ (Fig. 3a, b). The lower $E_\text {FA}^\text {SS}$ under high crosslinker turnover is in agreement with the observed lower $P_\text {BindingSites}(r)$. As $\chi$ increases, $\sum _{r={20}-{40} \textrm{nm}} P_\text {BindingSites}(r)$ at steady state decreases (Fig. 3d), suggesting that crosslinkers with higher turnover rates could not effectively reshape the actin network as those with lower turnover rates.

We found that $E_\text {FA}$ shows significant fluctuations even at a steady state (Fig. 3a). To measure the stability of contractility generation, we calculated the coefficient of variation (CV) of $E_\text {FA}$ at steady state($CV_\text {E, FA}^\text {SS}$), defined as the ratio between the standard deviation and the mean of the steady state energy. We saw that $CV_\text {E, FA}^\text {SS}$ decreases as the number of myosins and crosslinkers increases (Supplementary Fig. S2), suggesting that larger contractility corresponds to smaller fluctuations. The higher stability may be explained by the increased crosslinking. The tightly connected filament network generates a stronger mechanical structure that is less subjective to fluctuations. Similarly, a larger $\chi$ (faster turnover rate) reduces the steady-state energy but increases the relative fluctuation (Fig. 3c). We also noticed that $CV_\text {E, FA}^\text {SS}$ is negatively correlated with $E_\text {FA}^\text {SS}$ (Fig. 3e). Taken together, our results suggest that increasing the number of myosin motors, and crosslinkers, or decreasing the crosslinker turnover rate, enhances the contractility and lowers the relative mechanical fluctuations of a stress fiber.

### Tensile stretching enhances short-term contractility

**Figure 4:**
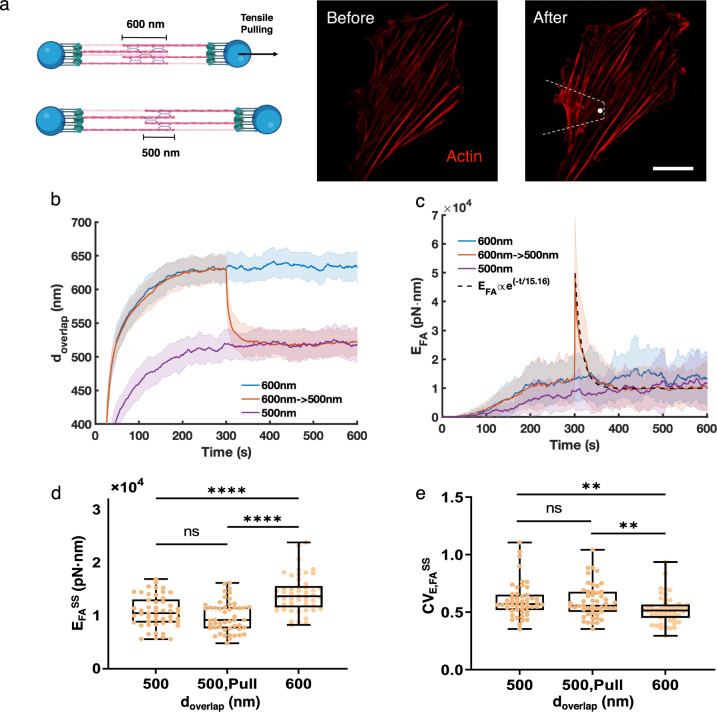
Tensile pulling regulates stress fiber contractility. (**a**, left) An illustration of the stress fiber before and after pulling in the simulation. Bundle overlap changes from $\sim$ 600 nm to $\sim$ 500 nm after pulling. (**a**, right) Vascular smooth muscle cells co-transfected with RhoA-EGFP constructs (green channel not shown) and actin-mRFP (red) were subjected to tensile mechanical stimulation using fibronectin functionalized probes. AFM-induced mechanical stimulation of the actin cytoskeleton was monitored by confocal imaging of live cells as previously described^30^. Images were acquired before and after a tensile mechanical stimulation experiment. The scale bar corresponds to 20 µm. Dashed lines represent the AFM cantilever above the cell. (**b**) The actin bundle overlap distance ($d_\text {overlap}$) as a function of time. Stress fibers were initialized with $d_\text {overlap} \sim$ 500  nm, $\sim$ 600   nm, and pulling from $d_\text {overlap}\sim$ 600   nm to $d_\text {overlap}\sim$ 500  nm, respectively. (**c**) The focal adhesion energy increases rapidly at the time of pulling, then relaxes to a new steady state. The relaxation curve is fitted to an exponential decay (RMSE $=$ 0.4). (**d,e**) The focal adhesion energy at steady state (**d**) and its fluctuations defined by CV (**e**) at $d_\text {overlap} \sim$ 500  nm, pulling from $d_\text {overlap}\sim$ 600  nm to $d_\text {overlap}\sim$ 500  nm, and $d_\text {overlap}\sim$ 600  nm, respectively. In the box plots, lower and upper boundaries represent the 25th and 75th percentiles, the line inside the box represents the median, and the lower and upper error lines represent the minimum and maximum values. Filled circles indicate all data points. T-tests were performed, ****$p<0.0001$ and **$p<0.01$. (**b–e**) $N_\text {Motor} = 48$, $N_\text {Crosslinker} = 240$, $\chi = 1$, and N $=$ 50 runs per condition.

Stress fibers can remodel under external axial stress, leading to changes in both cell mechanics and morphology^30,34^. To study how external mechanical stimuli affect the stress fiber contractility, we simulated the mechanical pulling or stretching experiment^30,34–37^ by moving one of the focal adhesion sites away from the other, increasing their distance (Fig. 4a, and Supplementary Video 2). We controlled the magnitude of external mechanical stimuli by varying the pulling distance ($d_{\text {pull}}$). This pulling process was broken down into multiple small, consecutive steps within 1 second (as was done in our previous work^8^). At $t=300 s$, upon 100nm pulling, the overlap between two actin bundles, $d_\text {overlap}$, changes from $\sim$600 nm to $\sim$500 nm (Fig. 4b). At the same time, $E_\text {FA}$ shows a significant increase, and gradually relaxes to a steady state value (Fig. 4c).

To understand how $d_\text {pull}$ affects stress fiber contractility, we first examined the short-term response. The rapid energy increase after pulling can be viewed as an elastic response of the actin bundle to pulling. Then the stress fiber slowly remodels and $E_\text {FA}$ decreases exponentially. Such exponential decay of $E_\text {FA}$ is in agreement with the viscoelastic behavior of the actomyosin network, where the energy relaxation is regulated by the dynamic remodeling of actin networks, including crosslinker turnover and myosin motors binding, unbinding, and walking. Due to large $E_\text {FA}$ variations, we computed the average $E_\text {FA}$ over 50 replicas ($\langle E_\text {FA} \rangle$) and fitted the energy decay to an exponential function (Fig. 4c).

$$\begin{aligned} { \langle E_\text {FA}(t) \rangle = \langle E_\text {FA}^\text {SS} \rangle + ( \langle E_\text {FA,peak} \rangle - \langle E_\text {FA}^\text {SS} \rangle ) e^{-t / \tau }, } \end{aligned}$$

where *t* is the time after pulling, $E_\text {FA,peak}$ is the peak energy immediately after pulling, and $\tau$ is the characteristic time scale for the energy decay. $\tau$ measures the time for the relative energy ($\langle E_\text {FA,peak} \rangle - \langle E_\text {FA}^\text {SS} \rangle$) to decay to $1/e \approx 0.37$ of the initial value. We suggest that $E_\text {FA,peak}$ and $\tau$ quantify the elastic and viscoelastic responses of the stress fiber, respectively. We found $\tau \sim {10}-{20}$ s, much longer than the pulling timescale and the turnover dynamics of crosslinkers and myosin motors. In MEDYAN simulation, the mechanical energy is minimized every millisecond. Therefore, the high value of $E_\text {FA}$ immediately after pulling is not due to unbalanced residual forces. In other words, the external rapid pulling induces a short-term enhancement of the stress fiber contractility $\sim {10}-{20}$ s, in agreement with previous experimental observations^30,36,37^.

We next examined whether $d_\text {pull}$ affects steady state contractility at a longer timescale. We noticed that after pulling, the value of $E_\text {FA}$ eventually decreased to a similar level as the energy in the stress fiber with $d_\text {overlap} \sim {500}$nm (Fig. 4c). This observation indicates that steady state contractility after pulling only depends on how much the two actin bundles overlap (Fig. 4d). Higher bundle overlap allows more myosin motors to bind and pull on actin filament pairs attached to different focal adhesion sites, which is more efficient in generating contractility than pulling on filament pairs from the same focal adhesion site. The enhancement of contractility is also partly due to crosslinkers localization, as more crosslinkers concentrate in the overlapping region when $d_\text {overlap}$ is higher (Supplementary Fig. S3). In the steady state, the stress fibers with higher overlap also displayed lower fluctuations (Fig. 4e), in agreement with the observation in Fig. 3e. We further examined the correlation between $d_\text {overlap}$ and contractility during stress fiber development. We found that contractility is also positively correlated with $d_\text {overlap}$ (Supplementary Fig. S4). Taken together, our results show that higher overlap leads to more crosslinkers and stronger contractile forces in the steady state, which is consistent at different pulling amplitudes, $d_\text {pull}$.

### The amplitude of pulling regulates short-term stress fiber mechanical response

**Figure 5:**
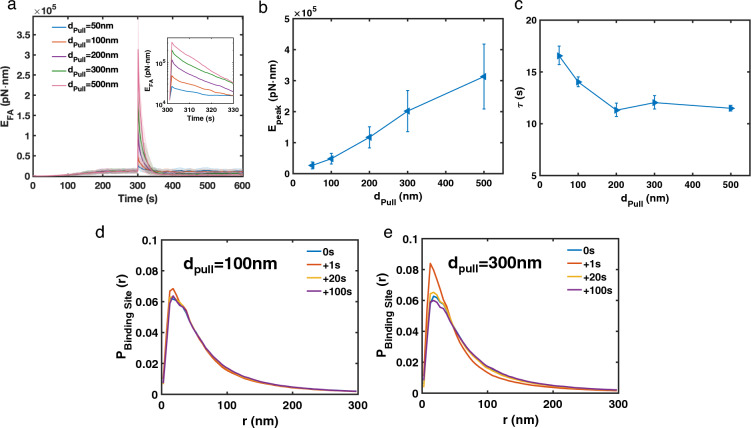
The magnitude of the external mechanical stimulus affects stress fiber mechanical response. (**a**) Focal adhesion energy as a function of time with varying pulling distance ($d_\text {pull}$). Insert shows the average focal adhesion energy from 300 to 330 s in a logarithmic scale. Shadings represent the 25th and 75th percentiles. (**b**) $E_\text {FA,peak}$ increases with increasing $d_\text {pull}$. Error bars represent the standard deviation from the mean. (**c**) $\tau$ decreases with the increasing $d_\text {pull}$. Error bars represent 95% confidence intervals for the fitting. (**d,e**) $P_\text {BindingSites}(r)$ right before pulling (0s) and at different time points after pulling (1 s, 20 s, and 100 s) with 100 nm pulling (**d**) and 300 nm pulling (**e**). (**a–e**) $N_\text {Motor} = 48$, $N_\text {Crosslinker} = 240$, $\chi = 1$, and N $=$ 50 runs per condition.

To understand how stress fibers respond to the magnitude of external mechanical stimuli, we varied $d_\text {pull}$ between the focal adhesion sites and examined the contractility change. $E_\text {FA,peak}$ increases as the $d_\text {pull}$ increases (Fig. 5a, b). If we consider the stress fiber as a viscoelastic material, then the increasing $E_\text {FA,peak}$ is due to the elastic response to the increasing deformation. Interestingly, we found that $\tau$ decreases as $d_\text {pull}$ is increased from 50 to 200 nm (Fig. 5c). Since $\tau$ should not depend on the magnitude of deformation for passive viscoelastic materials, we hypothesized that decreasing $\tau$ is due to changes in actin network remodeling. Indeed, while small pulling (small $d_\text {pull}$) has little impact on the distribution of crosslinker binding sites (Fig. 5d), larger pulling (larger $d_\text {pull}$) increases the crosslinker binding sites at $r \sim {20}$–40 nm (Fig. 5e). Such instantaneous changes provide more available binding sites for crosslinking, which could increase the network remodeling speed and reduce $\tau$. As the network remodels, $P_\text {BindingSites}(r)$ at $r \sim {20}$–40 nm decreases and eventually reaches a similar value as before pulling.

**Figure 6:**
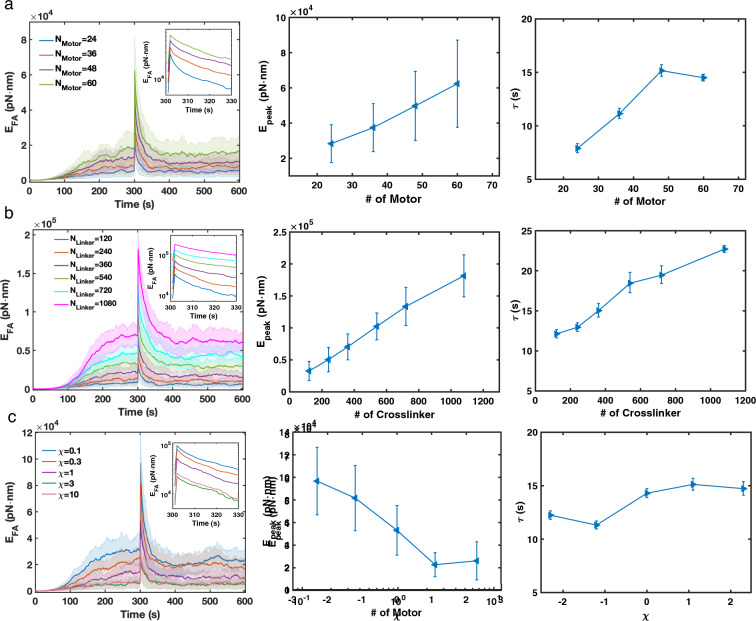
The focal adhesion energy relaxation is determined by the elastic and viscoelastic properties of the stress fiber. (**a–c**) Focal adhesion energy versus time (left), $E_\text {FA,peak}$ (middle), and $\tau$ (right) at varied the number of myosin motors (**a**), the number of crosslinkers (**b**), and the crosslinker turnover speed $\chi$ (**c**). (**a–c**, left panels) Shadings represent the 25th and 75th percentiles. Inserts show the average focal adhesion energy from 300 to 330 s in logarithmic scale. (**a–c**, middle panels) Error bars represent 95% confidence intervals for the fitting. (**a–c**, right panels) Error bars represent 95% confidence intervals for the fitting. (**a–c**) $N_\text {Motor} = 48$, $N_\text {Crosslinker} = 240$, and $\chi = 1$ unless noted in the figure. N $=$ 50 runs per condition.

We next examine how myosin motors and crosslinkers affect stress fiber contractility under tensile forces. We found that $E_\text {FA,peak}$ is positively correlated with the number of crosslinkers and myosins. On the other hand, $\tau$ increases as the concentration of crosslinkers or myosins increases, except at high myosin concentrations (Fig. 6a-b). We also found that crosslinker turnover rates affect $E_\text {FA,peak}$, but, surprisingly, have little impact on the energy relaxation rate (Fig. 6c). As $\chi$ increases, the energy immediately after pulling decreases, which can be explained by comparing the timescale of crosslinker turnover and the timescale of the pulling event. At low $\chi = 0.1$, the mean crosslinker turnover time is around 30 s, which is significantly longer than the pulling timescale (1 s). In this case, mechanical energy builds up during pulling. When the crosslinker turnover rate reaches $\sim$ 0.3 s ($\chi = 10$), energy cannot accumulate because crosslinkers could quickly unbind and release mechanical energy during pulling, resulting in a lower $E_\text {FA,peak}$. Although fast crosslinker turnover reduces $E_\text {FA,peak}$, it also decreases $P_\text {BindingSites}(r)$ at a small radius r (Fig. 3d), resulting in slower network remodeling. As a consequence, stress fibers with a higher $\chi$ have a lower $E_\text {FA,peak}$ as well as slower relaxation, making $\tau$ insensitive to crosslinker turnover. Taken together, our results suggest that myosin motors and crosslinkers regulate stress fiber formation in different ways, having a profound impact on both stress fiber contractility generation and stress fiber response to external mechanical stimuli.

## Discussion

How does the structural remodeling of stress fibers generate contractile force? In this report, we used the active matter simulation platform MEDYAN to study the role of myosin motors and crosslinkers in regulating contractility within individual stress fibers. Our model consisted of two interlocking actin bundles attached to focal adhesion complexes. We measured contractility in terms of contractile forces exerted on focal adhesion sites or energy stored between the stress fibers and the focal adhesion sites. While experimental approaches often examine the integrated mechanics of cells, including the extracellular matrix, focal adhesion, and other cytoskeletal networks, our model provides detailed molecular insights into the mechanics of individual stress fibers.

We found that stress fibers can optimize their contractility through structural remodeling that promotes crosslinker binding, although this ability is weaker when crosslinkers have a faster turnover rate. The steady-state contractility increases as the concentration of crosslinkers and myosin motors increase but decreases as the linker turnover rate increases. The relative fluctuations of steady-state contractility also decreases as the average adhesion energy increases. The contractility is also positively correlated with the size of the overlapping region between actin bundles. When subjected to external mechanical stimuli at focal adhesion sites, stress fibers exhibit viscoelastic properties and relax to a new steady state with a characteristic time of 10–20 s. This response depends on the concentrations of crosslinkers and myosin motors, the crosslinker turnover rate, and the magnitude of the applied mechanical stimulus.

We showed that crosslinker binding dynamics is an indispensable parameter for stress fiber contractility, and this regulation may also be explained from a thermodynamic perspective. In active systems like stress fibers, we can consider crosslinkers as a structural ratchet that hinders mechanical energy relaxation and sustains the mechanical energy when myosin motors dissociate from the actin filaments^13^. For crosslinkers with a faster turnover rate, the ratchet effect is weaker as the crosslinkers can quickly unbind and relax the mechanical energy stored in the actin network. As a result, a higher crosslinker turnover rate makes myosin motors less efficient and reduces contractility. Moreover, faster crosslinker turnover also helps to relax the mechanical energy induced by external mechanical stimuli, reducing the relaxation timescale $\tau$.

Crosslinker proteins, particularly $\upalpha$-actinin, are known to display the slip-bond behavior that larger tensile force enhances crosslinker unbinding^15–17^. Such behavior could reduce stress fiber contractility due to force-induced crosslinker unbinding^10,47^. Although the slip-bond of crosslinker was implemented in our simulation, its effect is limited in the range of forces applied in this work. Instead, we showed that contractility reduces with a higher crosslinker turnover rate without changes in the actin network connectivity. Taken together, these results emphasize the importance of actin organization and network remodeling in cytoskeletal force generation.

Our model captures the salient actomyosin network properties of a stress fiber and paves the way to better understand the cellular mechanical transduction. One limitation of this model is that the stress fibers in live cells could contain segments of actin bundles with alternating polarity^53^, which could potentially be studied using MEDYAN with multiple detached actin bundles. On the other hand, direct actin structure modifications and signaling cascades are simultaneously activated during mechanosensing. The signaling cascades could also alter actin structure by modulating filament polymerization, myosin activation, and crosslinker dynamics, creating complex feedback control on actomyosin structures and contractility. Future studies on how signalling cascades synergistically work with the direct restructuring of actin network under mechanical cues will provide new insights into cell biomechanics and mechanosensation.

## Methods

### MEDYAN simulations

Simulations in this work were performed in the active matter simulation platform Mechanochemical Dynamics of Active Networks (MEDYAN). The details about MEDYAN have been published previously^8,10,38,54–56^. Briefly, MEDYAN simulates reaction-diffusion systems using a variant of the Gillespie algorithm. Events like filament polymerization and depolymerization, crosslinking, and motor walking on the filaments are modeled as chemical reactions. These chemical events generate mechanical stress, computed as force fields for the polymer network. The chemistry simulation is coupled to a mechanical energy minimization process.

### Mechanical model and parameters

The actin filaments are modeled as cylinders connected at hinge points. Each full-sized cylinder has an equilibrium length of 108 nm, equivalent to 40 actin monomers. A quadratic energy penalty is applied if a cylinder deviates from its equilibrium length. The filament’s bending energy is computed at each hinge point, depending on the angle between the axial directions of the neighboring cylinders. The volume exclusion interaction between cylinders is used to prevent the filament from crossing through each other. Implementations of this potential have been discussed in detail previously^57^. Bound linking proteins, including $\upalpha$-actinin and non-muscle myosin II (NMII), are modeled as harmonic springs attached to binding sites on the filaments, with various equilibrium lengths and force constants. The actomyosin network is restricted within a volume using a repulsive boundary potential. However, cytoskeletal networks in the simulations for this work are far away from the simulation boundary, so the boundary effect is minimal. The barbed ends of the filaments are connected to the fixed focal adhesion sites using harmonic springs. Given the total mechanical energy of the system, the forces acting on bead positions are derived analytically. The conjugate gradient (CG) minimization algorithm is used to minimize this mechanical energy.

### Chemical model

Next Reaction Method^58^ is employed in MEDYAN to stochastically sample trajectories of the reaction-diffusion master equation. Small protein molecules such as G-actin monomers and unbound linking proteins are modeled as diffusing species and are tracked only by their molecule counts in each compartment. Apart from diffusion, the following events are also modeled as chemical reactions: filament polymerization and depolymerization, crosslinker binding and unbinding, as well as NMII walking on actin filaments. Reaction rates of certain reactions are further affected by local mechanical states. Specifically, under high tension, $\upalpha$-actinin proteins tend to unbind faster exhibiting slip-bond behavior, while NMII will unbind and walk more slowly exhibiting catch-bond and stalling behavior. These mechanosensitive behaviors have been previously discussed in detail^55^.

### Simulation setup

To simulate a stress fiber with endpoints attached at focal adhesion sites, we construct two antiparallel actin bundles with ends attached to two fixed focal adhesion sites (Fig. 1a). Each site is attached to the barbed ends of a bundle of 20 actin filaments using elastic springs. Initially, the distance between the two sites is 2750 nm, unless otherwise specified. Regardless of the forces applied, the positions of the sites are fixed throughout the simulation unless we manually move them. Each filament is initialized as a cylinder (40 actin monomers), with the minus end pointing towards the other site. We swap the filament polymerization dynamics between the barbed ends and the pointed ends, to ensure that the bundles roughly remain parallel during their initial growth phase. We also initialized the system with diffusing G-actin molecules, such that when the filament growth reaches the steady state (total polymerization propensity equals the total depolymerization propensity), the two actin bundles have an overlap of approximately 600 nm. Parameters for Myosin are those from non-muscle myosin II. Table 1 contains the default parameters used to generate the simulation results in this work. Unless otherwise specified, the parameters are the same as those previously published for actin cytoskeleton simulations using MEDYAN^38,55^.

**Table 1:** Default parameters for filament bundle simulations.

Description	Value
Number of actin filaments in each bundle	20
Initial number of cylinders in each actin filament	1
Reaction volume	${1} {\upmu }m \times {1} {\upmu }m \times {6} {\upmu }m$
Initial G-actin copy number	23,000
Initial NMII copy number	48
Initial $\upalpha$-actinin copy number	240
Linker turnover rate scaling factor ($\chi$)	1
Focal adhesion attachment equilibrium length	100 nm
Focal adhesion attachment spring constant	1 pN/nm

### The radial probability density function for binding site distances

To obtain $P_\text {BindingSites}(r)$, we simplify the problem by assuming that each hinge point in the filament represents a possible binding site for linkers. For a simulation snapshot, we collect all such hinge point locations $\{x_i\}$, and collect pairwise distances of these points in a list $\{d_I\}$, where

1 $$\begin{aligned} I \in \left\{ (i,j) \ \mid \ i< j, \text {hinge points} ~i~ \text{and}~j~ \text {are on different filaments} \right\} \end{aligned}$$

and

2 $$\begin{aligned} d_{(i,j)} = |x_i - x_j|. \end{aligned}$$

The resulting list of pairwise distances $\{d_I\}$ is used to estimate $P_\text {BindingSites}(r)$. $P_\text {BindingSites}(r)$ is normalized such that $\int 4\pi r^2 P_\text {BindingSites}(r) dr = 1$.

### Determining steady state

With the time trajectories of a certain quantity $\{q_i\}$ ($i=1,2,...,T$) sampled every second, we estimated the time entering the steady state ($t_0$) by minimizing the error for the estimator of the steady-state quantity $\langle q \rangle$^59^. The estimator ${\hat{q}}_{[t_0,T]}$ uses all the data starting at $t=t_0$:

3 $$\begin{aligned} {\hat{q}}_{[t_0,T]} = \frac{1}{T} \sum _{t=t_0}^T q(t). \end{aligned}$$

The overall expected error over all realizations of the time series $\{q_i\}$ is

4 $$\begin{aligned} \delta ^2 {\hat{q}}_{[t_0,T]} = {\mathbb {E}}\left[ ({\hat{q}}_{[t_0,T]} - \langle q \rangle )^2 \right] . \end{aligned}$$

To generate the steady-state starting times in Fig. 2a, we first assumed that the steady state is always reached starting $t={400}$ s and used all the samples after this time to find the “true steady state average” $\langle q \rangle$. For the ensemble expectation ${\mathbb {E}}[\cdot ]$, we used the average among all the time series.

### Data analysis and modeling fitting

Custom MATLAB and Julia scripts were written for data analysis and plotting. Box plots with scattering data points were generated using GraphPad PRISM7. To fit the result shown in Fig. 3e, we assumed a simple power law model and perform linear fitting of the data in logarithmic space using MATLAB. The fitting to the exponential decay model for FA energy was also conducted by linear fitting in logarithmic space using MATLAB.

### Cell culture and transfection

Vascular smooth muscle cells isolated from rat cremaster arterioles^60^, a gift from Dr. Michael Davis, Department of Medical Pharmacology and Physiology, University of Missouri, Columbia, MO, were cultured on fibronectin (Sigma, Saint Louis, MO) functionalized substrates. Then, cells were transfected with a green fluorescent protein (GFP) constructs using the Nucleofector apparatus (Amaxa, Gaithersburg, MD) with Nucleofector kit VPI-1004 as previously described^61^. The following fluorescent protein constructs were used: pcDNA3-EGFP-RhoA-wt (Addgene 12965) or vinculin-GFP (a gift from Kenneth Yamada, National Institute of Dental and Craniofacial Research, Bethesda, MD), and actin-mRFP (a gift from Michael Davidson, Florida State University, Tallahassee, FL). 

### Live cell imaging

The microscope system used for these studies combines a CSU-22 Yokogawa spinning-disk confocal scanning head (Yokogawa Electric Inc, Japan) with a Bioscope SZ atomic force microscope (AFM) from Bruker Instruments Inc (Santa Barbara, CA) mounted on top of an inverted Olympus IX-81 optical microscope equipped with a PLAN APO 60 $\times$ oil 1.45 NA objective lens^62^. Live VSM cells expressing GFP-constructs were imaged at 24 h after plating. Confocal images acquired as stacks of 20 planes with 0.25 $\upmu$m step size at an exposure time of 100 ms were further processed offline and presented as projections. Imaging experiments were performed in phenol-red free cell culture media, at room temperature.

### AFM mechanical stimulation

For tensile stress stimulation of vascular smooth muscle cells, the AFM probe consisting of a 2 µm glass bead functionalized with fibronectin. The AFM was used in contact imaging mode^63^. The AFM probe was placed on the cell surface and allowed to initiate the formation of a strong focal adhesion. The mechanical stimulation consisted of controlled upward movement of the AFM cantilever in discrete steps by applying tensile force in a range between $0.1-1 nN$ as previously described^30^. Imaging was performed after each tensile force application as presented above.

## Supplementary Information

Supplementary Figure S1.

Supplementary Figure S2.

Supplementary Figure S3.

Supplementary Figure S4.

Supplementary Video 1.

Supplementary Video 2.

Supplementary Legends.

## Data Availability

The modeling code is available in Digital Repository at the University of Maryland(DRUM): http://hdl.handle.net/1903/29500.
