# Supplementary figures and images for: Myosin and α-actinin regulation of stress fiber contractility under tensile stress
Source: Sci Rep. 2023 May 29;13:8662. doi: 10.1038/s41598-023-35675-7 (PMC10227020; doi:10.1038/s41598-023-35675-7)

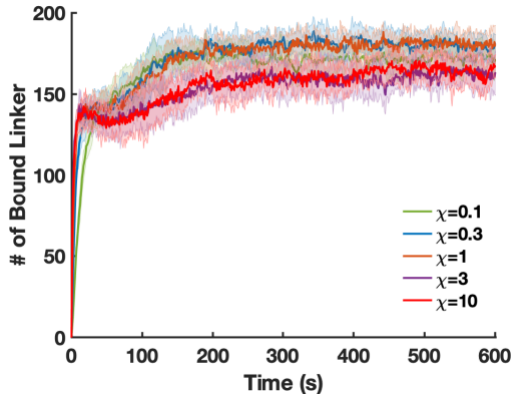

Supplement: Supplementary file 1 — Supplementary Figure S1. [file 41598_2023_35675_MOESM1_ESM.pdf]

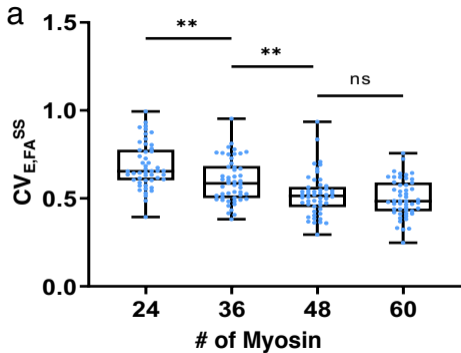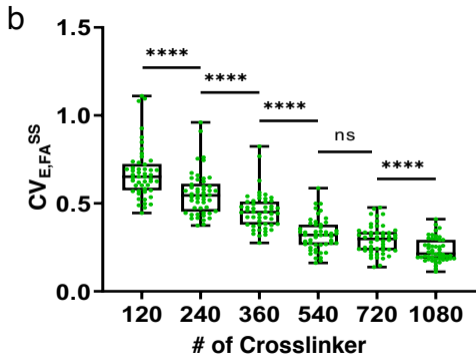

Supplement: Supplementary file 2 — Supplementary Figure S2. [file 41598_2023_35675_MOESM2_ESM.pdf]

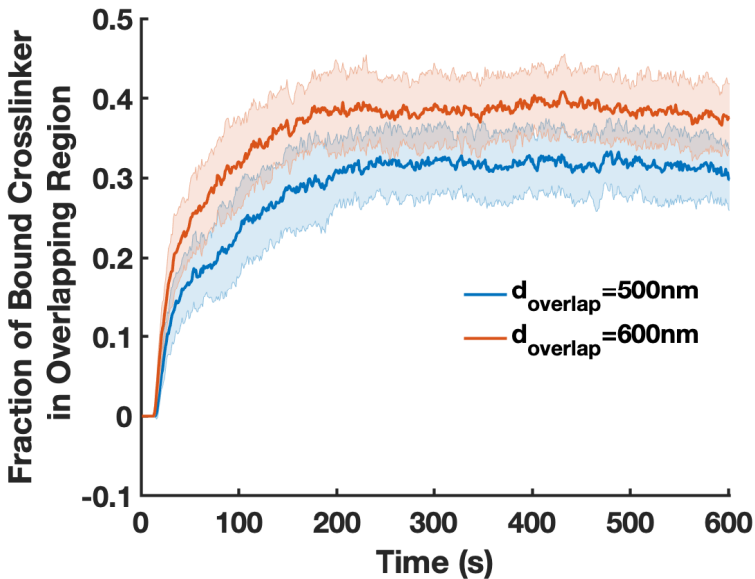

Supplement: Supplementary file 3 — Supplementary Figure S3. [file 41598_2023_35675_MOESM3_ESM.pdf]

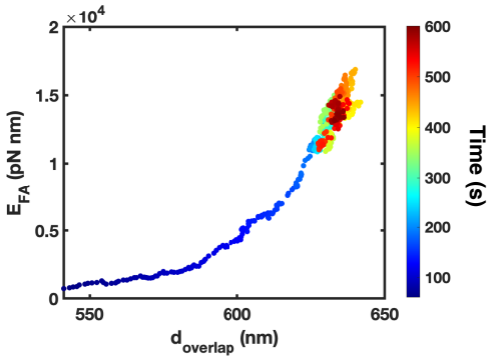

Supplement: Supplementary file 4 — Supplementary Figure S4. [file 41598_2023_35675_MOESM4_ESM.pdf]
