# Supplementary material for: Myosin and α-actinin regulation of stress fiber contractility under tensile stress
Source: Sci Rep. 2023 May 29;13:8662. doi: 10.1038/s41598-023-35675-7 (PMC10227020; doi:10.1038/s41598-023-35675-7)
Supplement: Supplementary file 7 — Supplementary Legends. [file 41598_2023_35675_MOESM7_ESM.pdf]

Supplementary Videos

Video 1. A representative video of actin stress fiber development. Actin filaments (red) attach to the focal adhesion sites (gray spheres), and myosin motors are blue, crosslinkers are green. The system was initialized with 20 short filaments at each focal adhesion site and a pool of free actin monomers. Actin filaments were allowed to polymerize based on experimentally measured parameters until reaching a kinetic steady state as shown in Fig. 2a.

Video 2. A representative video of stress fiber under tensile pulling. The focal adhesion site (gray spheres) on the left is pulled at $t = 300s$ after the stress fiber has reached a steady state. The locations of the focal adhesion site before and after pulling are marked. Actin filaments are red, and myosin motors are blue, crosslinkers are green.
